# State Space Formulation of Nonlinear Vibration Responses Collected from a Dynamic Rotor-Bearing System: An Extension of Bearing Diagnostics to Bearing Prognostics

**DOI:** 10.3390/s17020369

**Published:** 2017-02-14

**Authors:** Peter W. Tse, Dong Wang

**Affiliations:** Department of Systems Engineering and Engineering Management, City University of Hong Kong, Tat Chee Avenue, Hong Kong, China; dongwang4-c@my.cityu.edu.hk

**Keywords:** bearing prognostics, non-linear vibration responses, dynamic rotor-bearing system, remaining useful life, acceleration life testing

## Abstract

Bearings are widely used in various industries to support rotating shafts. Their failures accelerate failures of other adjacent components and may cause unexpected machine breakdowns. In recent years, nonlinear vibration responses collected from a dynamic rotor-bearing system have been widely analyzed for bearing diagnostics. Numerous methods have been proposed to identify different bearing faults. However, these methods are unable to predict the future health conditions of bearings. To extend bearing diagnostics to bearing prognostics, this paper reports the design of a state space formulation of nonlinear vibration responses collected from a dynamic rotor-bearing system in order to intelligently predict bearing remaining useful life (RUL). Firstly, analyses of nonlinear vibration responses were conducted to construct a bearing health indicator (BHI) so as to assess the current bearing health condition. Secondly, a state space model of the BHI was developed to mathematically track the health evolution of the BHI. Thirdly, unscented particle filtering was used to predict bearing RUL. Lastly, a new bearing acceleration life testing setup was designed to collect natural bearing degradation data, which were used to validate the effectiveness of the proposed bearing prognostic method. Results show that the prediction accuracy of the proposed bearing prognostic method is promising and the proposed bearing prognostic method is able to reflect future bearing health conditions.

## 1. Introduction

In most rotary machines, bearings are widely used in various industries to support the rotating shafts. Their failures accelerate failures of other adjacent components and result in unexpected machine breakdowns. Analyses of nonlinear vibration responses collected from a dynamic rotor-bearing system are widely used to diagnose different bearing faults [1]. A bearing consists of an outer race, an inner race, a cage, and several rollers. When there is a defect on the surface of either the outer race or the inner race, an impulse generated by a roller striking the defect is observed in a nonlinear vibration response collected from the casing of a dynamic rotor-bearing system [2,3,4,5]. Moreover, when a shaft rotates, more and more impulses are observed in the nonlinear vibration response. Experimental results have proved that these impulses are not purely periodic but slightly random, which causes difficulties of bearing diagnostics [6]. 

In recent years, many advanced methods have been proposed to analyze the nonlinear vibration responses caused by bearing faults [7,8,9]. One of the most attractive methods for bearing diagnostics is spectral kurtosis [10], which aims to calculate kurtosis values of signals obtained by different filters with different frequency supports. Based on the idea of spectral kurtosis, some improvements, such as the improved Kurtogram [11], the enhanced Kurtogram [12], the sparsogram [13], etc., have been designed to detect bearing faults in different cases. However, these methods [10,11,12,13] can only provide rough results for bearing fault diagnosis. To improve their visual inspection performances and achieve optimal filtering [14], different optimization algorithms, such as genetic algorithm [13], differential evolution, etc., and different objective metrics, such as kurtosis [15], entropy [16], smoothness index [17], sparsity measurement [13], etc., have been thoroughly investigated. 

Besides bearing diagnostics, bearing performance degradation assessment is an emerging and hot topic in recent years and attracts much attention. The bearing performance degradation assessment focuses on evaluations of the current bearing health condition [18] and its health evolution over time. In other words, the bearing performance degradation aims to assess how far the current bearing health condition deviates from a normal bearing health condition. Some methods, such as wavelet filtering [19,20], hidden Markov model [21], support vector data description [22], Gaussian mixture model [23], etc., have been proposed to track the current bearing health condition. However, the above methods belong to supervised learning. They require sufficient historical data to train the associated models. Therefore, their deficiencies are apparent when historical data are insufficient or unavailable. 

Recently, a novel earth mover distance-based bearing performance degradation assessment method [24] was proposed by one of the co-authors to describe bearing deterioration over time. Even though the aforementioned method is effective in evaluating the current health condition of bearings, it has some limitations, illustrated as follows. Firstly, artificial bearing defects were introduced to normal bearings prior to the bearing acceleration life testing. This means that the nonlinear vibration responses collected from the dynamic rotor-bearing system are not natural bearing degradation data, which may not well reflect bearing performance degradation from normal to failure. Secondly, for their proposed health index, it is assumed that one of the bearings used for supporting the shaft is in good condition. In other words, a pair of bearings does not degrade simultaneously. It is natural to argue that such an assumption is not completely true. Thirdly, their proposed method only focuses on evaluations of the current bearing health conditions rather than predictions of the future bearing health conditions and predictions of bearing remaining useful life (RUL). 

Particle filtering [25] has been used to solve any nonlinear state space models by using a number of random particles and their associated weights. The key step used in this kind of conventional particle filtering is the design of a proper importance function so as to update the weights of the random particles. Even though conventional particle filtering was used in fault prognosis for gas turbines [26], lithium ion batteries [27] etc., the proper procedure for designing the function to update the weights is still questionable. Unscented transform is a numerical method to find limited deterministic sigma points so as to estimate the mean and variance of a transformed variable. Aiming to target the shortcomings of conventional particle filtering, the use of unscented transform combined with particle filtering or the so-called unscented particle filtering was proposed [28]. 

According to our recent literature review in fault prognosis, the use of unscented particle filtering in bearing prognosis is seldom reported. This paper reports how unscented particle filtering could be tailor-made to predict the remaining useful life (RUL) of bearing. The results of the feasibility study have been stated in this paper. With the success of the feasibility study, several new contributions have been made to the research field of bearing prognosis. Firstly, a new setup for testing the bearing acceleration life was designed and the bearing’s degradation data was naturally collected from real running bearings. Secondly, based on the bearing degradation data, its nonlinear vibration responses were analyzed and then a bearing health indicator (BHI) was designed to indicate the severity of a damaged bearing. Thirdly, the state space formulation of the nonlinear vibration responses collected from a dynamic rotor-bearing system was designed to intelligently estimate the bearing’s RUL. That is, a state space model of the BHI was constructed to mathematically track the health evolutions of the BHI. With the help of the above methods and the verification results generated from the experiments, the proposed BHI was able to predict the RUL of real operating bearings with good accuracy.

The rest of this paper is organized as follows. In Section 2, the basic theories of the conventional particle filtering and unscented transform are introduced. In Section 3, the design of state space formulation of nonlinear vibration responses collected from a dynamic rotor-bearing system is reported. The procedures to intelligently predict the RUL of bearings are also discussed in this section. In Section 4, the experimental results are presented and verification is provided to demonstrate the effectiveness of the proposed bearing prognostic method. Conclusions are drawn in Section 5.

## 2. The Basic Theories of Unscented Particle Filtering 

### 2.1. Particle Filtering

Particle filtering [29] aims to use $N_{s}$ random particles ${\left\{x_{k}^{i}\right\}}_{i=1}^{N_{s}}$ and their associated weights ${\left\{\omega_{k}^{i}\right\}}_{i=1}^{N_{s}}$ to implement a recursive Bayesian filter and to characterize the posterior probability density function $p(x_{k}|z_{1:k})$. Here, $x_{k}$ is the system state and $z_{1:k}$ are measurements. Therefore, particle filtering provides a solution for solving a linear or nonlinear state space model. Ideally, it is expected to draw random particles from $p(x_{k}|z_{1:k})$, which can be approximated by:

(1) $$p(x_{k}|z_{1:k})\approx \sum_{i=1}^{N_{s}}\omega_{k}^{i}\delta (x_{k}-x_{k}^{i}),$$

where δ(⋅) is the Dirac delta function. In many cases, it is impossible to directly draw random particles from the true posterior density function $p(x_{k}|z_{1:k})$. Nevertheless, it is possible to draw random particles from another proposal distribution $q(x_{k}|z_{1:k})$ called an importance function. Suppose that the true posterior density function $p(x_{k}|z_{1:k})$ is proportional to an analytical function $\pi (x_{k}|z_{1:k})$. The weight of the $p(x_{k}|z_{1:k})$ can be represented by [29,30]:

(2) $$\omega_{k}^{i}\propto \frac{p(x_{k}^{i}|z_{1:k})}{q(x_{k}^{i}|z_{1:k})}\propto \frac{\pi (x_{k}^{i}|z_{1:k})}{q(x_{k}^{i}|z_{1:k})}.$$

If the importance function is factorized as [29,30]

(3) $$q(x_{k}^{i},x_{k-1}^{i}|z_{1:k})=q(x_{k}^{i}|x_{k-1}^{i},z_{1:k})q(x_{k-1}^{i}|z_{1:k-1})$$

and the $p(x_{k}^{i}|z_{1:k})$ is represented by [29,30],

(4) $$p(x_{k}^{i}|z_{1:k})\propto p(z_{k}|x_{k}^{i})p(x_{k}^{i}|x_{k-1}^{i})p(x_{k-1}^{i}|z_{1:k-1}),$$

the weight $\omega_{k}^{i}$ can be iteratively updated by:

(5) $$\omega_{k}^{i}\propto \frac{p(x_{k}^{i}|z_{1:k})}{q(x_{k}^{i}|z_{1:k})}\propto \frac{p(z_{k}|x_{k}^{i})p(x_{k}^{i}|x_{k-1}^{i})p(x_{k-1}^{i}|z_{1:k-1})}{q(x_{k}^{i}|x_{k-1}^{i},z_{1:k})q(x_{k-1}^{i}|z_{1:k-1})}\propto \omega_{k-1}^{i}\frac{p(z_{k}|x_{k}^{i})p(x_{k}^{i}|x_{k-1}^{i})}{q(x_{k}^{i}|x_{k-1}^{i},z_{1:k})}.$$

If $q(x_{k}^{i}|x_{k-1}^{i},z_{1:k})=q(x_{k}^{i}|x_{k-1}^{i},z_{k})$ is satisfied, calculation of the weight $\omega_{k}^{i}$ can be simplified as:

(6) $$\omega_{k}^{i}=\omega_{k-1}^{i}\frac{p(z_{k}|x_{k}^{i})p(x_{k}^{i}|x_{k-1}^{i})}{q(x_{k}^{i}|x_{k-1}^{i},z_{k})}/\left(\sum_{i=1}^{N_{s}}\omega_{k-1}^{i}\frac{p(z_{k}|x_{k}^{i})p(x_{k}^{i}|x_{k-1}^{i})}{q(x_{k}^{i}|x_{k-1}^{i},z_{k})}\right).$$

In terms of Equation (6), the importance function depends on the previous system state $x_{k-1}^{i}$ and the current measurement $z_{k}$. However, in many applications of particle filtering, to simplify Equation (6), the importance function is usually chosen as a prior state transition function, namely $q(x_{k}^{i}|x_{k-1}^{i},z_{k})=p(x_{k}^{i}|x_{k-1}^{i})$. Then, Equation (6) is simplified as:

(7) $$\omega_{k}^{i}=\omega_{k-1}^{i}p(z_{k}|x_{k}^{i})/\left(\sum_{i=1}^{N_{s}}\omega_{k-1}^{i}p(z_{k}|x_{k}^{i})\right).$$

The major disadvantage of Equation (7) is that the current measurement is not considered in the importance function to update the weight $\omega_{k}^{i}$. Therefore, it is necessary to incorporate the current measurement to the importance function.

### 2.2. Unscented Transform

Unscented transform [25] aims to find limited deterministic sigma points to estimate the mean and variance of a transformed variable z=f(x). Here, x is a multivariate Gaussian distribution with *n*-dimensional mean vector m and n×n covariance matrix P, and f(⋅) is a linear or nonlinear function. The major difference between the particle filtering and the unscented transform is that an amount of random particles are used in particle filtering, whereas 2*n* + 1 deterministic sigma points are used in unscented transform. Simply speaking, the unscented transform can sufficiently provide a rough solution for solving any linear or nonlinear state space models. The working principle of the unscented transform is introduced as follows [25]:

Step 1. Determine 2*n* + 1 sigma points:

(8) $$\begin{array}{l} \chi^{0}=m,\\ \chi^{i}=m+\sqrt{n+\lambda }{\left[\sqrt{P}\right]}_{i},\\ \chi^{i+n}=m-\sqrt{n+\lambda }{\left[\sqrt{P}\right]}_{i},\;i=1,\ldots ,n, \end{array}$$

where ${\left[\cdot \right]}_{i}$ takes the *i-*th column of the matrix. $\lambda =3\alpha^{2}-n$ is the parameter to control the spread of the sigma points around the mean. Moreover, $\sqrt{P}{\sqrt{P}}^{T}=P$. 

Step 2. Propagate the determined sigma points via a non-linear function:

(9) $$Y^{i}=f(\chi^{i}),\;i=0,\ldots ,2n,$$

where $Y^{i}$ is called the transformed sigma points.

Step 3. Estimate the mean and covariance of the transformed variable by:

(10) $$E[g(x)]=u_{U}=\sum_{i=0}^{2n}W_{i}^{m}Y^{i},$$

(11) $$\mathrm{Cov}[g(x)]=S_{U}=\sum_{i=0}^{2n}W_{i}^{c}(Y^{i}-u_{U}){\left(Y^{i}-u_{U}\right)}^{T},$$

where $W_{i}^{m}$ and $W_{i}^{c}$ are respectively defined as:

(12) $$\begin{array}{l} W_{0}^{m}=\frac{\lambda }{\lambda +n},\\ W_{0}^{c}=\frac{\lambda }{\lambda +n}+(1-\alpha^{2}+\beta ),\\ W_{i}^{m}=\frac{1}{2\left(\lambda +n\right)},\;i=1,\ldots ,2n,\\ W_{i}^{c}=\frac{1}{2\left(\lambda +n\right)},\;i=1,\ldots ,2n, \end{array}$$

where β controls the prior information of x. According to [25], α and β are recommended to be the default values 1 and 0, respectively. 

## 3. State Space Formulation of Nonlinear Vibration Responses for the Bearing Prognostics of a Dynamic Rotor-Bearing System

**Figure 1:**
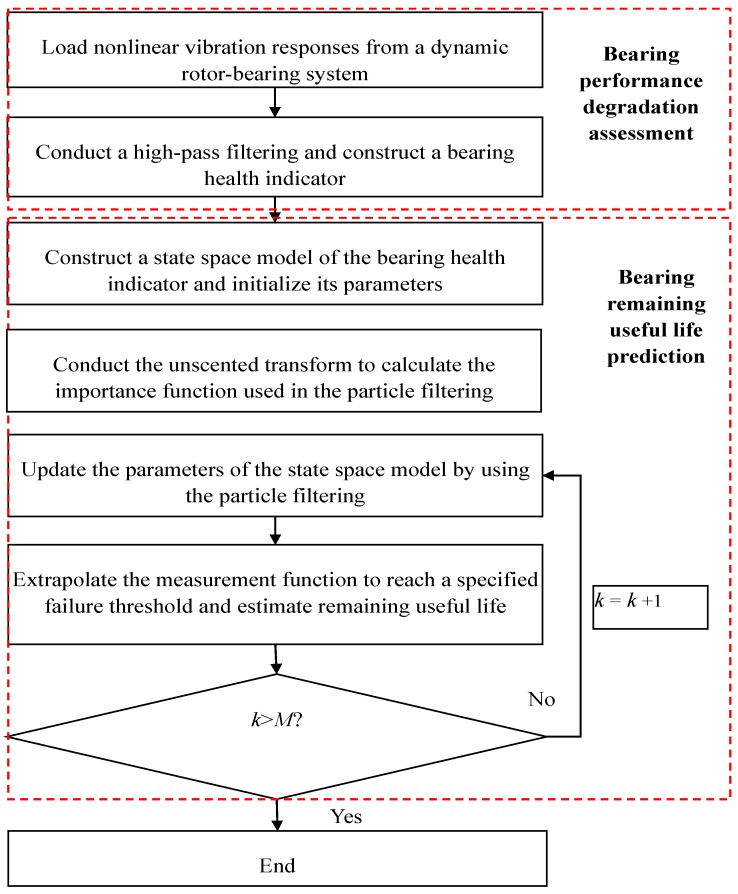
A flowchart of the proposed bearing prognostic method for prediction of bearing remaining useful life (RUL).

In this section, state space formulation of nonlinear vibration responses collected from a dynamic rotor-bearing system is built to predict bearing RUL. A flowchart of the proposed bearing prognostic method is given in Figure 1. The procedure of the proposed bearing prognostic method is summarized as follows. Firstly, analyses of the nonlinear vibration responses should be conducted to extract a bearing degradation trend. This means that a BHI should be found to describe the current health condition of the bearing. Secondly, a state space model of the BHI is constructed to mathematically track the health evolutions of the BHI. Finally, bearing RUL is predicted using unscented particle filtering. The details of the above steps are introduced in the following sections.

### 3.1. Bearing Performance Degradation Assessment

**Figure 2:**
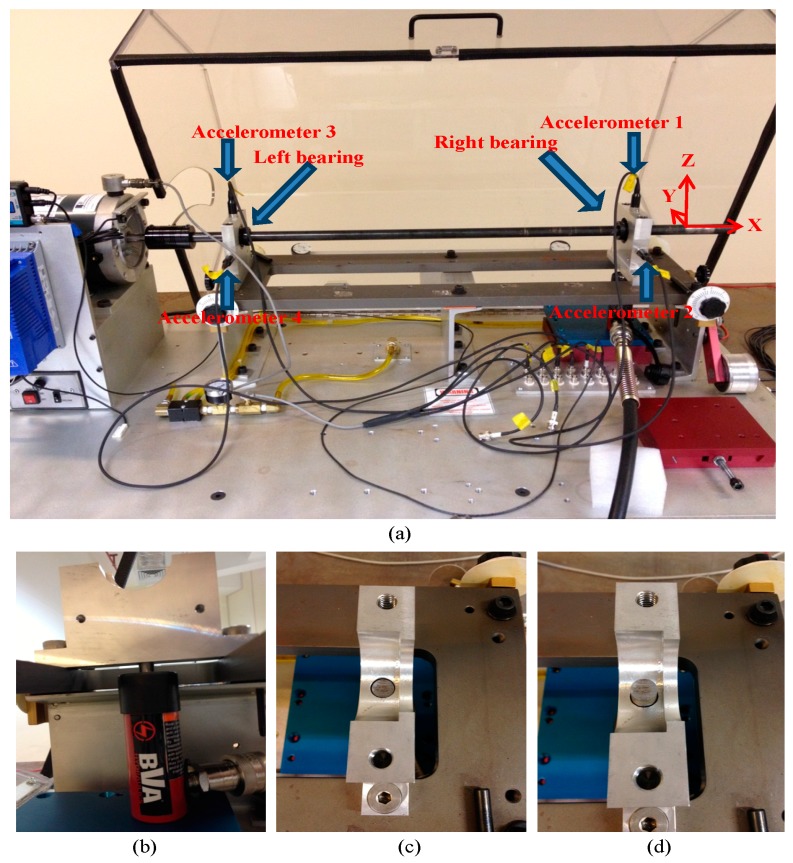
The experiment setup: (**a**) the locations of four accelerometers; (**b**) the application of the hydraulic jack to the outer race of the bearing; (**c**) the hydraulic jack without touching the surface of the bearing; and (**d**) the hydraulic jack applied to the surface of the outer race.

Prior to bearing performance degradation assessment, a new bearing acceleration life testing is introduced. The aim of the acceleration life testing is to collect natural bearing degradation data. The experiments of bearing prognosis were designed by the first author. The experiment setup, shown in Figure 2a, is a rotary machine testing platform called “Machinery Fault Simulators (MFS)” that is manufactured by SpectraQuest Inc. (Richmond, VA, USA). The MFS is designed for conducting experimental work to reveal the signatures of common machinery faults without compromising production schedule or profits. This bench-top system has a larger baseplate, a powerful driver as a motor, various gearboxes, bearings, belt drives, reciprocating mechanisms, induction motors, pumps, and compressors. Then, various faults can be introduced either individually or jointly in a totally controlled environment. The types of faults can be rotor balancing, alignment, resonance, bearing defects, crack shafts, fan and mechanical rub, pump defects, etc.

Four accelerometers were attached to the dynamic rotor-bearing system. The sensitivities of the accelerometers from 1 to 4 are 0.093 V/g, 0.095 V/g, 0.093 V/g, and 0.092 V/g, respectively. Accelerometers 1 and 2 were used to measure nonlinear vibration responses from the vertical and horizontal directions of the right bearing. Accelerometers 3 and 4 were used to measure nonlinear vibration responses from the vertical and horizontal directions of the left bearing. Specifications of the right and left bearings and associated bearing fault characteristic frequencies are listed in Table 1. During the experiment, the running speed of the motor or the shaft rotation frequency was set to 30 ± 0.5 Hz. The sampling frequency was set to 36,864 Hz, or collecting 36,864 vibration samples per second. The length of each measurement collected from each of the four accelerometers was set to 1.0 s. Measurements were collected every 30 min until the bearings generated loud sound and failed. A total of 145 vibration measurements was collected from each accelerometer or a total of 5,345,280 vibration samples was collected from each accelerometer. For the four accelerometers, the number of vibration samples collected was 21,381,120 samples. Since the correlation between vibration and rotor position can be affected by disturbances in the spindle speed [31,32], in order to obtain a steady operating condition, the motor rotating speed or the shaft rotation frequency was set to 30 ± 0.5 Hz.

To accelerate bearing life from a normal health condition to failure, a hydraulic jack with a continuously applied force was exerted to a location of the outer race of the right bearing through an adapter. When the hydraulic jack was working or applied to the surface of the bearing’s outer race, a force of 128 kg (20 kg/cm^2^) was applied to the bottom of that bearing’s outer race. That is, the hydraulic jack acted as an external force continuously exerted on the bearing so as to generate a wear status to the bearing. The schematic working principle of the hydraulic jack with the adapter is shown in Figure 2b–d. Moreover, the right bearing was running without proper lubricant. The experiment stopped until the rotor-bearing system had heavily vibrated and created a loud noise. There were 145 timely measurements collected until the shutdown of the rotor-bearing system. In this paper, the vibration data collected from accelerometer 1 were used as this bearing suffered from extra external force generated by the hydraulic jack and continuous wear in that location of the bearing was expected.

**Figure 3:**
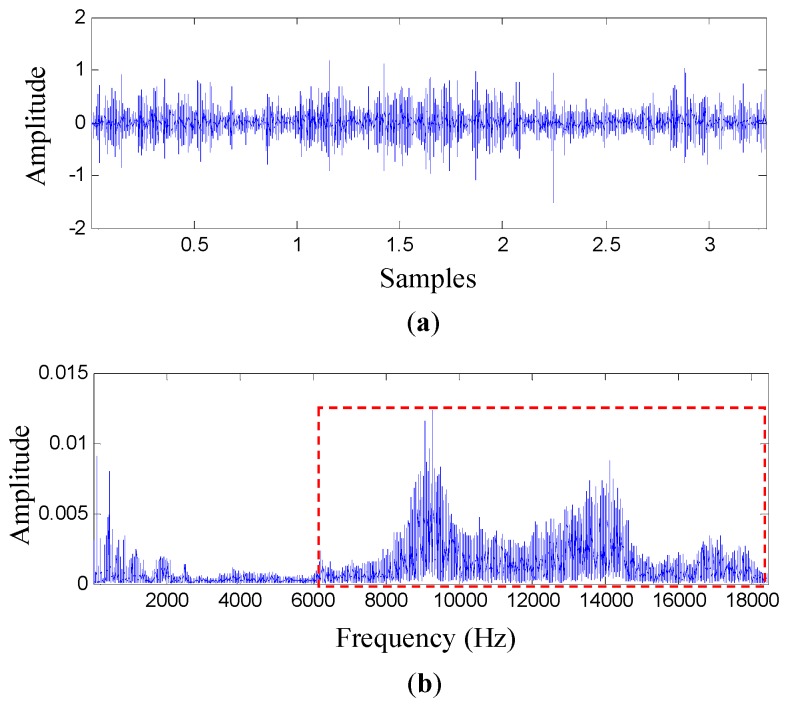
A vibration measurement collected from the vertical direction of the casing of the right bearing at measurement number 50: (**a**) its temporal signal and (**b**) its corresponding frequency spectrum.

It is known that impacts generated by bearing defects excite resonant frequencies of structures and adjacent components, and they induce modulation phenomenon. For bearing fault data produced by a single artificial defect, it is more likely to use some advanced signal processing methods to extract bearing fault features, such as bearing fault characteristic frequencies, as shown in Table 1. For natural bearing degradation data, bearing fault types are unknown and multiple bearing faults may occur simutaneously. In our preliminary analyses, some advanced signal processing methods, such as the fast Kurtogram and the improved Kurtogram, fail to provide any information related to bearing faults. Considering these points, it is preferable to use a high-pass filter to retain some resonant frequency bands and to remove the interruptions from low-frequency vibration components. A vibration measurement collected from the vertical direction of the casing of the right bearing at measurement number 50 and its corresponding frequency spectrum are plotted in Figure 3a,b, respectively. Note that a high-pass filter with a cutoff frequency of 6000 Hz to 18,000 Hz was used to retain the high-frequency components generated by the tested bearing. That is, the high-frequency range from 6000 Hz to 18,000 Hz, which contains high vibration energy, was retained and analyzed.

**Figure 4:**
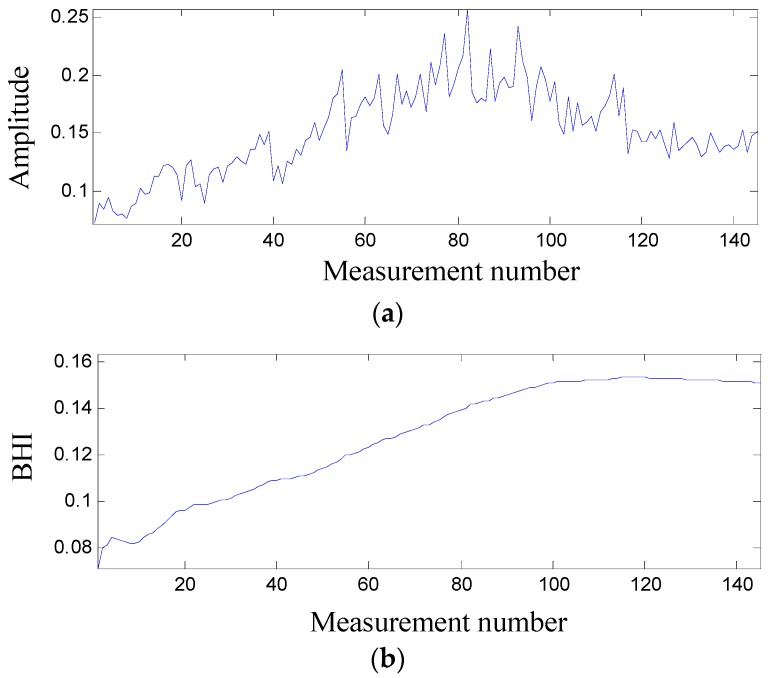
Bearing degradation assessment: (**a**) the RMS after applying the high-pass filter; (**b**) the degradation trend generated by the BHI.

Since the statistical root mean square (RMS) can characterize the raw degradation data in the view of vibration energy, the RMS is used as an important fault indicator. If faults have occurred in a bearing, the generated vibration energy is expected to be much higher than that from a normal running bearing. The measured RMS was preprocessed by a high-pass filter and its result is shown in Figure 4a, in which it is shown that the RMS fluctuates so much that it is not easy to use for bearing prognostics. Consequently, it is necessary to construct a BHI that has a direct connection with the RMS. In terms of the central tendency of the RMS, a BHI is defined as follows:

(13) $$\mathrm{BHI}(j)=\frac{\sum_{k=1}^{j}\mathrm{RMS}(k)}{j},\;j=1,2,\ldots ,145,$$

where RMS(*k*) is the *i*-th RMS after the high-pass filter was conducted on a vibration signal stored at the *i*-th measurement number. The BHI is plotted in Figure 4b, in which it can be seen that the BHI increases continuously and sharply until it reached measurement number 100. After that, the increase rate of the BHI was slower than before. This is because, when the tested bearing was suffering from the continuous high force exerted by the hydraulic jack, wear was created [31,32]. 

Initially only small spall or cracks would be formed, but the damage to the surface became severe over time. Continuously applying such force to the same location would create wear to the bearing at an outer race, balls and then an inner race and a cage. That is, all parts at this point of the bearing would be damaged in time. Moreover, once the balls were damaged, they would cause other parts of the bearing to be damaged when the damaged balls rotated to the other part of the bearing. At a later stage, the outer race, the inner race, the balls, and the cage were all worn at different degrees. The conditions would deteriorate over time with a continuous increase in vibration energy, as shown in Figure 4b. At a certain point, because of the continuous rubbing of different surfaces of the bearing, some of the damaged surfaces became smoother than before, causing the rate of increasing in vibration energy to be slower or the slope of degradation trend becomes flat. In Figure 4b, it is seen that the proposed BHI can monitor the current health conditions of the right bearing. 

### 3.2. State Space Formulation of Bearing Performance Degradation

To predict the future health conditions of the right bearing, its state space model should be built. Firstly, it is necessary to find a mathematical model to properly describe the BHI. In Figure 4b, it seems that measurement number 116 is the peak of the trend created by BHI. Such a degradation trend is the typical bearing wear trend.

Because the maximum of the BHI is 0.1537 at measurement number 116, the failure threshold of the BHI could be set to 0.1537. Moreover, the effective bearing degradation length is from 1 to 116. By using goodness of fit, the R-squared values of a linear polynomial function, a quadratic polynomial function, and an exponential function are 0.9866, 0.9925, and 0.9663, respectively. The closer the value of the R-squared is to 1, the better the performance of the function. Consequently, the quadratic polynomial function is used as the measurement function of the bearing state space model. Then, the bearing state space model used in this paper is constructed as follows:

(14) $$a_{k}=a_{k-1}+v_{1}$$

(15) $$b_{k}=b_{k-1}+v_{2}$$

(16) $$c_{k}=c_{k-1}+v_{3}$$

(17) $${\mathrm{BHI}}_{k}=f(k)+v_{4}=a_{k}\times k^{2}+b_{k}\times k+c_{k}+v_{4},$$

where $a_{k}$, $b_{k}$, and $c_{k}$ are the parameters of the BHI. $v_{1}$, $v_{2}$, $v_{3}$, and $v_{4}$ are the additive Gaussian noises with zero means and different standard deviations $\sigma_{1},\sigma_{2},\sigma_{3}$, and $\sigma_{4}$, respectively. Initializations of Equations (14) to (17) are clarified at the beginning of Section 4. At last, $\sigma_{1}^{2},\sigma_{2}^{2},\sigma_{3}^{2}$ form a state noise covariation matrix Q, in which the diagonal elements are $\sigma_{1}^{2},\sigma_{2}^{2},\sigma_{3}^{2}$, respectively.

### 3.3. Posterior State Parameter Estimation of the Bearing State Space Model Using Unscented Particle Filtering

According to the bearing state space model and the fundamental theories of particle filtering and unscented transform, when a new BHI is available, the three state parameters $a_{k}$, $b_{k}$, and $c_{k}$ can be iteratively updated by the following steps.

Step 1. Draw initial random particles ${\left\{a_{0}^{i}\right\}}_{i=1}^{N_{s}}$, ${\left\{b_{0}^{i}\right\}}_{i=1}^{N_{s}}$, and ${\left\{c_{0}^{i}\right\}}_{i=1}^{N_{s}}$ from the initial distributions of $q(a_{0})=N(a_{0},\sigma_{1}^{2})$, $q(b_{0})=N(b_{0},\sigma_{2}^{2})$, and $q(c_{0})=N(c_{0},\sigma_{3}^{2})$, respectively. If $N_{s}$ is taken to be large then we will be estimating the true likelihood quite precisely, but its computing will be very expensive. On the other hand, a small value of $N_{s}$ will result in cheap evaluations, but low computation burden. Here, $N_{s}$ is equal to 1000, which is manually selected and is deemed to be sufficient for our research’s purpose. Set all initial weights ${\left\{\omega_{0}^{i}\right\}}_{i=1}^{N_{s}}$ to a value of $1/N_{s}$. Suppose that the initial distribution for the use of the unscented transform is $p(x_{0}^{i})=p(a_{0}^{i},b_{0}^{i},c_{0}^{i})$ ≈ $N(a_{0}^{i},b_{0}^{i},c_{0}^{i}|\;m_{0},P_{0})$. Initializations of the $m_{0}$ and the $P_{0}$ are clarified in Section 4. According to the theory of the unscented transform, only seven sigma points (2×3+1) are required to propagate via the measurement function:

(18) $$\begin{array}{l} \chi_{0}^{0}=m_{0},\\ \chi_{0}^{j}=m_{0}+\sqrt{3}{\left[\sqrt{P_{0}+Q}\right]}_{j},\\ \chi_{0}^{j+2}=m_{0}-\sqrt{3}{\left[\sqrt{P_{0}+Q}\right]}_{j},\;j=1,2,3 \end{array}$$

(19) $$Y_{0}^{j}=f(\chi_{0}^{j}),\;j=0,\ldots ,6.$$

The predicted mean $u_{U}^{0}$, the predicted covariance $S_{U}^{0}$, and the cross-covariance $C_{U}^{0}$ of the state and the measurement are calculated as follows:

(20) $$E[g(x_{0})]=u_{U}^{0}=\sum_{j=0}^{6}W_{j}^{m}Y_{0}^{j}$$

(21) $$\mathrm{Cov}[g(x_{0})]=S_{U}^{0}=\sum_{j=0}^{6}W_{j}^{c}(Y_{0}^{j}-u_{U}^{0}){\left(Y_{0}^{j}-u_{U}^{0}\right)}^{T}+v_{4}^{2}$$

(22) $$\mathrm{Cov}[x_{0},g(x_{0})]=C_{U}^{0}=\sum_{j=0}^{6}W_{j}^{c}(\chi_{0}^{j}-m_{0}){\left(Y_{0}^{j}-u_{U}^{0}\right)}^{T}.$$

When the first BHI is available, the $p(a_{1}^{i},b_{1}^{i},c_{1}^{i}|{\mathrm{BHI}}_{1})$ is approximated by the multivariate Gaussian distribution with the following parameters:

(23) $$m_{1}=m_{0}+C_{U}^{0}{\left(S_{U}^{0}\right)}^{-1}\left({\mathrm{BHI}}_{1}-u_{U}^{0}\right)$$

(24) $$P_{1}=P_{0}-C_{U}^{0}{\left(S_{U}^{0}\right)}^{-1}S_{U}^{0}{\left(C_{U}^{0}{\left(S_{U}^{0}\right)}^{-1}\right)}^{T},$$

where $C_{U}^{0}{\left(S_{U}^{0}\right)}^{-1}$ is the filter gain and can be determined by Equations (21) and (22). 

The importance function $q(x_{k}^{i}|x_{k-1}^{i},z_{k})$ used in Equation (6) is replaced with the joint posterior probability density function $p(a_{1},b_{1},c_{1}|{\mathrm{BHI}}_{1})$. Then, the weight updating formula is rewritten as:

(25) $$\omega_{1}^{i}=\frac{\omega_{0}^{i}\frac{p(z_{1}|a_{1}^{i},b_{1}^{i},c_{1}^{i})p(a_{1}^{i}|a_{0}^{i})p(b_{1}^{i}|b_{0}^{i})p(c_{1}^{i}|c_{0}^{i})}{p(a_{1}^{i},b_{1}^{i},c_{1}^{i}|{\mathrm{BHI}}_{1})}}{\sum_{i=1}^{N_{s}}\omega_{0}^{i}\frac{p(z_{1}|a_{1}^{i},b_{1}^{i},c_{1}^{i})p(a_{1}^{i}|a_{0}^{i})p(b_{1}^{i}|b_{0}^{i})p(c_{1}^{i}|c_{0}^{i})}{p(a_{1}^{i},b_{1}^{i},c_{1}^{i}|{\mathrm{BHI}}_{1})}}.$$

Compared with the weight updating shown in Equation (7), Equation (25) considers the first BHI when the importance function is used. Then, the posterior probability density functions of the three parameters are respectively represented by:

(26) $$p(a_{1}|{\mathrm{BHI}}_{1})\approx \sum_{i=1}^{N_{s}}\omega_{1}^{i}\delta (a_{1}-a_{0}^{i})$$

(27) $$p(b_{1}|{\mathrm{BHI}}_{1})\approx \sum_{i=1}^{N_{s}}\omega_{1}^{i}\delta (b_{1}-b_{0}^{i})$$

(28) $$p(c_{1}|{\mathrm{BHI}}_{1})\approx \sum_{i=1}^{N_{s}}\omega_{1}^{i}\delta (c_{1}-c_{0}^{i}).$$

To avoid the degeneracy problem of the original particle filtering, which is that most of the weights become negligible after a few iterations, the systematic resampling is required to redrawn random particles from the posterior probability density functions calculated by the original particle filtering according the size of $\omega_{1}^{i}$ if the ${\left(\sum_{i=1}^{N_{s}}{\left(\omega_{1}^{i}\right)}^{2}\right)}^{-1}$ is smaller than half of the *N_s_*.

Step 2. Draw $N_{s}$ random particles ${\left\{a_{k-1}^{i}\right\}}_{i=1}^{N_{s}}$, ${\left\{b_{k-1}^{i}\right\}}_{i=1}^{N_{s}}$, and ${\left\{c_{k-1}^{i}\right\}}_{i=1}^{N_{s}}$ from Equations (26)–(28), respectively. Suppose that the posterior probability density function $p(x_{k-1}^{i}|{\mathrm{BHI}}_{1:k-1})=p(a_{k-1}^{i},b_{k-1}^{i},c_{k-1}^{i}|{\mathrm{BHI}}_{1:k-1})$ ≈ $N(a_{k-1}^{i},b_{k-1}^{i},c_{k-1}^{i}|m_{k-1},P_{k-1})$ at iteration *k* − 1 is estimated by using the unscented transform. Only seven sigma points are required to propagate via Equation (17):

(29) $$\begin{array}{l} \chi_{k-1}^{0}=m_{k-1},\\ \chi_{k-1}^{j}=m_{k-1}+\sqrt{3}{\left[\sqrt{P_{k-1}+Q}\right]}_{j},\\ \chi_{k-1}^{j+2}=m_{k-1}-\sqrt{3}{\left[\sqrt{P_{k-1}+Q}\right]}_{j},\;j=1,2,3 \end{array}$$

(30) $$Y_{k-1}^{j}=f(\chi_{k-1}^{j}),\;j=0,\ldots ,6.$$

The predicted mean $u_{U}^{k-1}$, the predicted covariance $S_{U}^{k-1}$, and the cross-covariance $C_{U}^{k-1}$ of the state and the measurement are calculated as follows:

(31) $$E[g(x_{k-1})]=u_{U}^{k-1}=\sum_{j=0}^{6}W_{j}^{m}Y_{k-1}^{j}$$

(32) $$\mathrm{Cov}[g(x_{k-1})]=S_{U}^{k-1}=\sum_{j=0}^{6}W_{j}^{c}(Y_{k-1}^{j}-u_{U}^{k-1}){\left(Y_{k-1}^{j}-u_{U}^{k-1}\right)}^{T}+v_{4}^{2}$$

(33) $$\mathrm{Cov}[x_{k-1},g(x_{k-1})]=C_{U}^{k-1}=\sum_{i=0}^{6}W_{i}^{c}(\chi_{k-1}^{i}-m_{k-1}){\left(Y_{k-1}^{i}-u_{U}^{k-1}\right)}^{T}.$$

When a new BHI is available, the $p(a_{k}^{i},b_{k}^{i},c_{k}^{i}|{\mathrm{BHI}}_{1:k})$ is approximated by a multivariate Gaussian distribution with the following parameters:

(34) $$m_{k}=m_{k-1}+C_{U}^{k-1}{\left(S_{U}^{k-1}\right)}^{-1}\left(BHI_{k}-u_{U}^{k-1}\right)$$

(35) $$P_{k}=P_{k-1}-C_{U}^{k-1}{\left(S_{U}^{k-1}\right)}^{-1}S_{U}^{k-1}{\left(C_{U}^{k-1}{\left(S_{U}^{k-1}\right)}^{-1}\right)}^{T}.$$

The weight updating formula is rewritten as:

(36) $$\omega_{k}^{i}=\frac{\omega_{k-1}^{i}\frac{p(z_{k}|a_{k}^{i},b_{k}^{i},c_{k}^{i})p(a_{k}^{i}|a_{k-1}^{i})p(b_{k}^{i}|b_{k-1}^{i})p(c_{k}^{i}|c_{k-1}^{i})}{p(a_{k}^{i},b_{k}^{i},c_{k}^{i}|{\mathrm{BHI}}_{1:k})}}{\sum_{i=1}^{N_{s}}\omega_{k-1}^{i}\frac{p(z_{k}|a_{k}^{i},b_{k}^{i},c_{k}^{i})p(a_{k}^{i}|a_{k-1}^{i})p(b_{k}^{i}|b_{k-1}^{i})p(c_{k}^{i}|c_{k-1}^{i})}{p(a_{k}^{i},b_{k}^{i},c_{k}^{i}|{\mathrm{BHI}}_{1:k})}}.$$

If ${\left(\sum_{i=1}^{N_{s}}{\left(\omega_{k}^{i}\right)}^{2}\right)}^{-1}$ is smaller than half of *N_s_*, the systematic resampling is conducted to redrawn random particles from the posterior probability density function according to the size of $\omega_{k}^{i}$.

Step 3. Increase *k* to *k* + 1 and repeat Step 2 until k>M is satisfied. Here, *M* is the length of the available BHI. Then, the posterior probability density functions of the three parameters at iteration *M* can be respectively represented by:

(37) $$p(a_{M}|{\mathrm{BHI}}_{1:M})\approx \sum_{i=1}^{N_{s}}\omega_{M}^{i}\delta (a_{M}-a_{M}^{i})$$

(38) $$p(b_{M}|{\mathrm{BHI}}_{1:M})\approx \sum_{i=1}^{N_{s}}\omega_{M}^{i}\delta (b_{M}-b_{M}^{i})$$

(39) $$p(c_{M}|{\mathrm{BHI}}_{1:M})\approx \sum_{i=1}^{N_{s}}\omega_{M}^{i}\delta (c_{M}-c_{M}^{i}).$$

#### 3.4. Bearing Remaining Useful Life Prediction

Once the posterior probability density functions of the three parameters are estimated, extrapolations of the measurement function to a specified failure threshold, such as 0.1537 used in this paper, are used to calculate bearing RUL at measurement number *M*:

(40) $$\mathrm{RUL}(M)=\sum_{i=1}^{N_{s}}\omega_{M}^{i}\delta (\mathrm{RUL}-\inf(kk\in \mathrm{int}:a_{M}^{i}\times kk^{2}+b_{M}^{i}\times kk+c_{M}^{i}\geq 0.1537)+kk),i=1,2,\ldots ,N_{s},$$

where the “int” is the set of all integers and the “inf” takes the greatest lower bound of a set and *kk* is a future possible measurement number. The 50th, 5th, and 95th percentiles of Equation (40) can be regarded as the estimated RUL and its lower and upper bounds.

## 4. A Case Study of Bearing Prognostics

In this section, nonlinear vibration responses collected from the bearing acceleration life testing are used to illustrate how the proposed bearing prognostic method works and to validate its effectiveness in predicting bearing RUL. If more historical BHI data are available, the initial estimates of a, b, and c were calculated using the nonlinear least squares regression on historical BHI data. If historical BHI data are not available, the nonlinear least squares regression on part of the available BHI can be conducted. In this paper, based on the BHI from 1 to 20, the initial estimates of a, b, and c were calculated as −1.81 × 10^−6^, 9.11 × 10^−4^, and 7.635 × 10^−2^, respectively. The 95% confidence bounds of the initial estimates of a, b, and c were calculated as (−2.189 × 10^−6^, −1.431 × 10^−6^), (8.654 × 10^−4^, 9.569 × 10^−4^) and (7.519 × 10^−2^, 7.751 × 10^−2^), respectively. Therefore, according to six sigma principle, the standard deviations of the three parameters were artificially set to 1.2633 × 10^−7^, 1.5250 × 10^−5^, and 3.8333 × 10^−4^, respectively. Because the scale of the BHI is small, the standard deviation of the measurement noise was set to 0.005, which results in a small residual in Equation (17).

**Figure 5:**
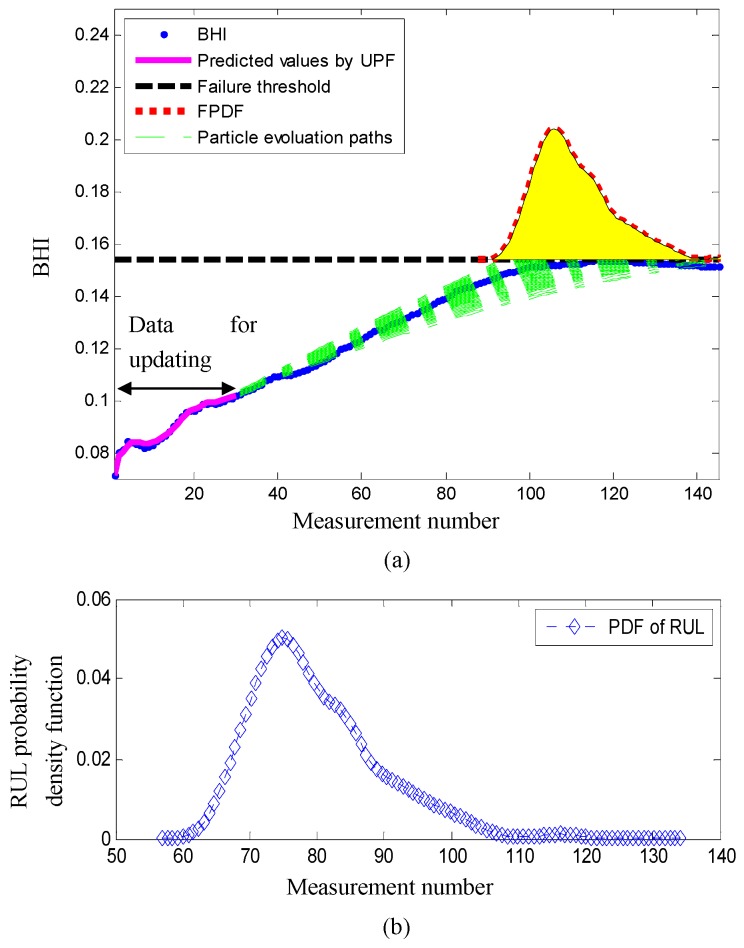
Bearing RUL prediction by using the proposed bearing prognostic method at measurement number 30. (**a**) The degradation trend; (**b**) the probability density function (PDF) of the RUL.

At measurement number 30, the predictions of the RUL are plotted in Figure 5. In Figure 5a, it is observed that the predictive values obtained by the unscented particle filtering are well matched with the true BHI. This means that the predictions obtained by the unscented particle filtering are suitable for tracking the BHI. The extrapolations of the measurement function are highlighted by the green dashed lines, which indicate the future health evolutions of the BHI. The probability density function of the RUL is plotted in Figure 5b, where the 5th, 50th, and 95th percentiles of the RUL are 68, 78, and 86 measurement numbers, respectively. The error between the estimated RUL 78 and the actual RUL 86 is eight measurement numbers. 

**Figure 6:**
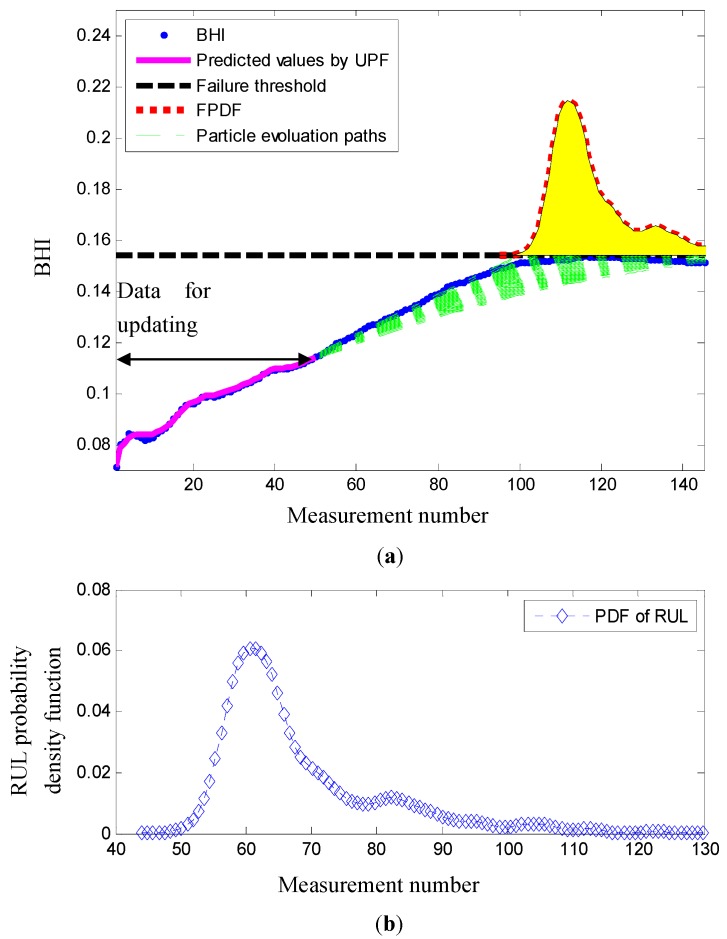
Bearing RUL prediction by using the proposed bearing prognostic method at measurement number 50. (**a**) The degradation trend; (**b**) the probability density function (PDF) of the RUL.

At measurement number 50, the predictions obtained by using the proposed bearing prognostic method are presented in Figure 6. In Figure 6a, it is observed that the predictive BHI obtained by the unscented particle filtering is very close to the true BHI. In other words, unscented particle filtering results in high prediction accuracies for the BHI. In Figure 6b, the probability density function of the RUL is plotted. It is found that the 5th, 50th, and 95th percentiles of the RUL are 56, 64, and 95 measurement numbers, respectively. The error between the actual RUL 66 and the predicted RUL 64 is two measurement numbers. 

To reduce the length of this paper, more bearing RUL predictions at document numbers from 20 to 110 with an increment of 10 are tabulated in Table 2, where the proposed bearing prognostic method results in the high prediction accuracies of the RUL.

In the standard particle filtering, the importance function is chosen as a prior state transition function and the calculation of the weights is largely simplified. In other words, the recent BHI is not considered in the importance function. To highlight the advantage of the unscented particle filtering, some comparisons are made by replacing the unscented particle filtering with the standard particle filtering in our proposed prognostic method. The results obtained by the standard particle filtering are tabulated in Table 3. From the prediction errors shown in Table 2 and Table 3, it is found that the prediction errors obtained by the standard particle filtering are larger than the prediction errors obtained by the unscented particle filtering, especially after file number 20. It should be noted that the unscented transform is not considered for comparisons because the unscented transform is just a method that can be used to make fast and approximate parameter distributions using a few deterministic sigma points, while the particle filtering uses a number of random particles to infer parameter distributions and is able to provide higher prediction accuracy.

## 5. Conclusions

The state space formulation of the nonlinear vibration responses collected from the dynamic rotor-bearing system was reported in this paper for the extensions from bearing diagnostics to prognostics. Prior to prediction of the RUL of bearing, the bearing performance degradation assessment was conducted by constructing the BHI. This indicator was then used to monitor the current health conditions of the bearing. Then, to mathematically describe the future health evolutions of BHI, the state space model of the BHI was constructed. Unscented particle filtering was introduced to calculate the posterior probability density functions of the parameters of the state space model. The extrapolations of the measurement function to the specified failure threshold were used to estimate the RUL of the inspected bearing. To validate the effectiveness of the proposed methods, a new setup for monitoring the bearing operational life was designed. Vibration data related to the deterioration of the bearing were collected naturally from real running bearings. The predicted results of the RUL demonstrated that the bearing prognostic method reported here is effective at inferring future bearing health conditions and the RUL of bearing. Moreover, comparisons with the conventional particle filtering were made to demonstrate that the method of unscented particle filtering can achieve better prediction accuracy than conventional particle filtering methods.

## Figures and Tables

**Table 1:** Specifications of bearings used in our experiment.

Specifications of Bearings	Parameters
Bearing model	MB ER-12K
Number of rolling elements	8
Rolling element diameter	7.9375 mm
Pitch diameter	33.4772 mm
Contact angle	0°
Fundamental train frequency (FTF)	11.3 Hz
Ball pass frequency outer (BPFO)	91.4 Hz
Ball pass frequency inner (BPFI)	148.5 Hz
Ball spin frequency (BSF)	59.8 Hz

**Table 2:** The predictions of the RUL by the proposed bearing prognostic method (Unit: measurement number).

Prediction at File Number	5th Percentile of Predicted RUL	50th Percentile of Predicted RUL	95th Percentile of Predicted RUL	Actual RUL	Error between Actual RUL and 50th Percentile of Predicted RUL
20	67	73	83	96	23
30	68	78	95.5	86	8
40	58	69	90	76	7
50	56	64	95	66	2
60	38	47	71	56	9
70	25	28	33	46	8
80	29	30	32	36	6
90	19	24	37	26	2
100	10	14	20	16	2
110	2	3	4	6	3

**Table 3:** The predictions of the RUL by replacing the unscented particle filtering with the standard particle filtering in our proposed prognostic method (Unit: measurement number).

Prediction at File Number	5th Percentile of Predicted RUL	50th Percentile of Predicted RUL	95th Percentile of Predicted RUL	Actual RUL	Error between Actual RUL and 50th Percentile of Predicted RUL
20	66	73	85	96	23
30	65	76	100	86	10
40	51	61	82	76	15
50	52	63	89	66	3
60	37	46	69	56	10
70	31	37	58	46	9
80	19	25	35	36	11
90	11	15	22	26	11
100	5	8	16	16	8
110	2	3	7	6	3
